# *In vitro* interaction of *Stenotrophomonas maltophilia* with human monocyte-derived dendritic cells

**DOI:** 10.3389/fmicb.2015.00723

**Published:** 2015-07-16

**Authors:** Emanuela Roscetto, Laura Vitiello, Rosa Muoio, Amata A. Soriano, Vita D. Iula, Antonio Vollaro, Eliana De Gregorio, Maria R. Catania

**Affiliations:** ^1^Dipartimento di Medicina Molecolare e Biotecnologie Mediche, Università Federico II, Napoli, Italy; ^2^Laboratorio di Immunologia Cellulare e Molecolare, Istituto di Ricovero e Cura a Carattere Scientifico, San Raffaele Pisana, Rome, Italy; ^3^DAI Medicina di Laboratorio, Azienda Ospedaliera Universitaria Federico II, Napoli, Italy

**Keywords:** DC maturation markers, cytokines secretion, cystic fibrosis, opportunistic pathogen, innate immune response

## Abstract

*Stenotrophomonas maltophilia* is increasingly identified as an opportunistic pathogen in immunocompromised, cancer and cystic fibrosis (CF) patients. Knowledge on innate immune responses to *S. maltophilia* and its potential modulation is poor. The present work investigated the ability of 12 clinical *S. maltophilia* strains (five from CF patients, seven from non-CF patients) and one environmental strain to survive inside human monocyte-derived dendritic cells (DCs). The effects of the bacteria on maturation of and cytokine secretion by DCs were also measured. *S. maltophilia* strains presented a high degree of heterogeneity in internalization and intracellular replication efficiencies as well as in the ability of *S. maltophilia* to interfere with normal DCs maturation. By contrast, all *S. maltophilia* strains were able to activate DCs, as measured by increase in the expression of surface maturation markers and proinflammatory cytokines secretion.

## Introduction

The environmental Gram-negative bacillus *Stenotrophomonas maltophilia* is increasingly identified as an opportunistic nosocomial pathogen for susceptible individuals, including immunocompromised, cancer, and cystic fibrosis (CF) patients (Brooke, 2012; Waters et al., 2013). Cases of community-acquired *S. maltophilia* infections have also been reported (Falagas et al., 2009b; Chang et al., 2014). Molecular analyses have revealed considerable heterogeneity among *S. maltophilia* isolates, including those coming from just one hospital (Roscetto et al., 2008; Kaiser et al., 2009; Rocco et al., 2009).

The intrinsic resistance to a wide spectrum of antibiotics (Crossman et al., 2008; Brooke, 2014), the ability to adhere and form biofilm on medical devices surfaces and airway epithelial cells (Pompilio et al., 2010), make *S. maltophilia* responsable of persistent nosocomial infections. *S. maltophilia* can cause various serious infections but it is most commonly associated with pneumonia (Weber et al., 2007; Chang et al., 2014; Chawla et al., 2014). *S. maltophilia* pneumonia may be complicated by persistent bacteremia associated with a significant increased mortality (Elsner et al., 1997; Falagas et al., 2009a).

Little is known about pathogenesis of *S. maltophilia*, and the genomic diversity exhibited by clinical isolates makes difficult the identification of pathogenic traits. Factors likely involved in the pathogenesis of *S. maltophilia* have been described (Chhibber et al., 2008; Pompilio et al., 2011; Roscetto et al., 2012). The ability to adhere and penetrate inside airway epithelial cell lines (Pompilio et al., 2010), and stimulate robust cytokine production in macrophages and monocytes are key steps in *S. maltophilia* infections (Waters et al., 2007; Härtel et al., 2013). Little is known on the interactions between *S. maltophilia* and dendritic cells (DCs). Immature DCs (iDCs) are sentinel cells found in skin, respiratory and gastrointestinal mucosae constantly recruited from blood to peripheral tissues, even in the absence of inflammatory processes (Guermonprez et al., 2002). Upon capture of soluble antigens or bacteria, iDCs undergo maturation, a profound change in gene expression transforming them from antigen-capturing to mature antigen-processing and -presenting cells (Guermonprez et al., 2002; Villadangos and Health, 2005).

Mature DCs migrate to secondary lymphoid organs to prime naïve T cells differentiation into effector T cells (Reis e Sousa, 2006). DCs produce interleukin-12p70 (IL-12p70), which activates the differentiation of T helper 1 (Hsieh et al., 1993) and cytotoxic T cells, and IL-1 beta, IL-6, and IL-23, required for differentiation of Th17 cells (Acosta-Rodriguez et al., 2007). Pathogenic bacteria have developed strategies which enable them to escape phagocytosis and killing by DCs, or affect DC maturation (Bueno et al., 2012).

The aim of this study was to compare the ability of *S. maltophilia* strains from CF and non-CF patients to survive in human monocyte-derived DCs, and evaluate whether isolates from CF patients have unique properties. We also examined the effect of *S. maltophilia* strains on the maturation of DCs, monitoring the expression of specific maturation markers by flow cytometry.

## Materials and Methods

### Bacterial Strains and Culture Conditions

Thirteen non-clonal clinical isolates of *S. maltophilia* were included in the study. Strains were isolated from diverse clinical settings, including CF and non-CF respiratory specimens, as well as non-respiratory specimens, collected at the Laboratory of Clinical Microbiology, University Federico II of Naples. The sequenced strain K279a was kindly provided by M. B. Avison (University of Bristol, Bristol, UK). The environmental strain LMG11104 was obtained from the BCCM (Belgium Coordinated Collections of Microorganisms/Laboratorium) voor Microbiologie Ghent, University of Ghent, Belgium. Details of the bacterial strains used in this study are given in Table 1. Bacteria were identified as *S. maltophilia* by biochemical characteristics and antibiotic resistance analysis, using the VITEK II system (bioMerieux) and confirmed by MALDI TOF MS (Bruker). Bacteria were grown overnight at 37°C for 24 h in Brain-Hearth Infusion broth (BHI; Becton Dickinson), and aliquots were frozen in BHI-glycerol at –80°C until use.

**Table 1:** ***Stenotrophomonas maltophilia* strains used in DC infection assay**.

**Strain name**	**Source**	**Swimming motility**	**Twiching motility**	**Biofilm production**
CF1545	Sputum (P)	++	+	++
CF781	Sputum (G)	++	+	++
CF635	Sputum (P)	–	–	–
CF2315	Sputum (G)	+	+++	++
CF1445	Pharyngeal aspirate (P)	+	++	++
NCF879	Sputum (ICU)	+++	+++	+++
NCF1489	Sputum (H)	+++	+++	+++
NCF1732	Bronchial aspirate (ICU)	+++	+++	+++
NCF1376	Bronchial aspirate (ICU)	++	++	+
NCF2035	Bronchial aspirate (ICU)	++	+++	+++
TFE66	Foot wound swab (D)	+++	++	+++
K279a	Blood infection	++	+	++
LMG11104	Tuberous roots	++	++	++

P, Pediatrics; G, Geriatrics; ICU, Intensive Care Unit; H, Hematology; D, Diabetology; LMG-labeled strain is from the Laboratorium voor Microbiologie Gent Culture Collection, Belgium. (+, weak; ++, moderate; +++, strong).

### Motility Assays

Swimming motility was evaluated on plates 1% tryptone, 0.5% NaCl containing 0.3% agar. A colony of bacteria was inoculated with a sterile toothpick placed directly into the center of the agar. After incubation for 48 h at 30°C, the diameters of the swimming motility zones were measured. Twitching motility was evaluated on plates 1% tryptone, 0.5% yeast extract, 0.5% NaCl containing 1% agar. A colony of bacteria was inoculated deep into the agar with a sterile toothpick at the bottom of twitch plates. After incubation at 37°C for 24 h, the zone of motility at the agar-petri dish interface was measured by staining with 1% crystal violet.

### Biofilm Assay

Biofilm formation was tested in 96-well microtiter plates according to Stepanovic et al. (2000). Briefly, one colony of the overnight cultures of bacterial strains was diluted in trypticase soy broth (TSB) in order to adjust the turbidity of the bacterial suspension to 0.5 McFarland standard, approximately 10^8^ CFU (colony forming units)/mL. Bacterial suspension was diluted 1:100 in TSB and 200 μL of the diluted bacteria was added to each well. Negative control wells contained broth only. The plates were incubated aerobically for 48 h at 37°C. Thereafter, the content of each well was aspirated and the wells washed three times with 300 μl of sterile phosphate buffered saline (PBS). Biofilm was fixed incubating microtiter plates for 1 h at 60°C. The plates were stained with 30 μl per well of 1% crystal violet for 15 min and washed with 150 μl of sterile PBS. The dye bound to the adherent cells was resolubilized with 150 μl of 95% ethanol per well. The optical density of each well was measured at 630 nm using a microplate reader (Biorad). Based on the optical densities of bacterial biofilms, all strains were classified into the following categories: no biofilm producers (–), weak (+), moderate (+), or strong (+++) biofilm producers, as previously described (Stepanovic et al., 2000).

### *In vitro* Generation and Culture of Human DCs

Immature dendritic cells were generated from peripheral blood mononuclear cells, as described (Sallusto and Lanzavecchia, 1994). Briefly, peripheral blood mononuclear cells were obtained from 30 ml of leukocyte-enriched buffy coat from healthy donors by centrifugation on a Ficoll-Hypaque plus (GE Healthcare) through density gradient. Monocytes were purified by positive selection using anti-CD14 mAb-conjugated magnetic microbeads (Miltenyi Biotec). CD14^+^ cells were cultured in RPMI 1640 supplemented with 10% fetal calf serum (Invitrogen), and 2 mM glutamine (Invitrogen) containing 50 ng/ml GM-CSF (granulocytes monocytes-colony stimulating factor) and 250 ng/ml IL-4 (Immunotools). Cells were cultured for 5–7 days in 5% CO_2_ atmosphere to obtain a population of iDCs. Purity of generated DCs was checked by flow cytometry, using Phycoerythrin (PE) conjugated anti-CD1a antibody (Becton Dickinson) on a FACScalibur flow cytometer (Becton Dickinson). DC populations were used when CD1a expression was > 95%. Written informed consent was obtained from each donor at the time of venous peripheral blood donation, in accordance with the Declaration of Helsinki, as approved by Azienda Ospedaliera Universitaria Federico II. All the experiments done by using blood donations were performed and analyzed anonymously, without any biographical reference to donors.

### DC Infection Assays

iDCs were seeded at a concentration of 10^6^ cells per well into 24-wells culture plates, infected with 2 × 10^7^ CFU/ml of mid-log growth phase *S. maltophilia* cells to yield bacterium-to-dendritic cell ratio of 20:1 and incubated for 1 h at 37°C in 5% CO_2_.

To assess the number of intracellular bacteria, the culture medium was gently removed, cells were washed twice with sterile PBS and further incubated for 1 h in fresh medium containing 600 μg/ml gentamicin or 1 mg/ml ceftazidime to kill extracellular bacteria. DCs were subsequently washed and incubated in 10 mM EDTA, 0.25% Triton X-100 (Sigma) for 5 min to release internalized bacteria. Lysates were serially diluted 1:10 and plated on TSA (Becton Dickinson) to quantify the number of viable intracellular bacteria. Entry Index (EI), i.e., the percentage (mean) of the inoculated CFUs that were internalized by iDCs after 1 h of infection, was determined as follows: EI = (CFU ml^–1^ recovered from DCs after 1 h of infection/CFU ml^–1^ applied to DCs) × 100.

To measure intracellular replication, after infection of DCs with *S. maltophilia* and antibiotic treatment cells were extensively washed and fresh medium was added. Incubation was continued for additional 16 h. After washing three times with PBS, intracellular bacteria were released by addition of 10 mM EDTA, 0.25% Triton X-100 and the number of CFU was determined by plating 10-fold dilutions on TSA. Intracellular Replication Index (RI), i.e., the percentage (mean) of the internalized CFUs that survived by iDCs after 18 h of infection, was determined as follows: RI = (CFU ml^–1^ recovered from DCs after 18 h of infection/CFU ml^–1^ recovered from DCs after 1 h of infection) × 100.

The bactericidal activity of gentamicin or ceftazidime at the used concentrations had been previously assessed. The effectiveness of drugs during infection assays was tested by supernatant sampling before DCs lysis and plating on TSA (data not shown).

### DCs Maturation Analysis

Dendritic cells were seeded in 24-well plates at a concentration of 10^6^ cells/ml and then exposed to RPMI 1640 only, 1 mg/ml LPS or live bacteria at an MOI of 20:1. After 18 h of incubation at 37°C and 5% CO_2_, DCs were harvested, washed, re-suspended in FACS buffer and DC maturation was then analyzed measuring CD86 and CD80 expression by flow cytometry, using FITC-conjugated anti-CD80 antibody and a PE-conjugated anti-CD86 antibody (Becton Dickinson).

For TNFα expression, cells were cultured for 2 h with the bacterial strains in the presence of 5 mg/ml Brefeldin A (Sigma) and then stained with anti-TNFα antibody (Becton Dickinson) using Cytofix/Cytoperm method (Becton Dickinson), according to manufacturer’s instructions. IL -12p70 production by DCs was measured from supernatants using OptEIA ELISA kits (Becton Dickinson), according to manufacturer’s instructions.

### Statistical Analysis

All assays were performed in three independent experiments. Paired Student’s *t*-test was used to evaluate the statistical differences between uninfected-DCs and *S. maltophilia*-infected DCs and between CFUs recovered from DCs after 1 h and after 17 h of incubation for each analyzed strain. Statistical differences between multiple groups of *S. maltophilia* strains were assessed using a Mann-Whitney non-parametric test. Values of *p* < 0.05 were taken as statistically significant differences.

## Results

### Internalization and Intracellular Replication of *S. maltophilia* in Immature Monocytes-derived Dendritic Cells

We have selected 13 strains from diverse clinical setting (Table 1). Some were isolated from the respiratory tract of CF patients (group 1, CF strains), others from the respiratory tract of non-CF patients (group 2, NCF strains), the remaining from non-respiratory specimens (group 3, NR strains). Motility is crucial both to colonize an environment and to induce formation of structured surface-associated communities of bacteria called biofilm. For each strain, both motility and the ability to form biofilm in polystyrene microtiter plates were analyzed. All CF and NR strains exhibited reduced swimming and twiching motilities compared to NCF strains (Table 1). Biofilm forming ability greatly varied among strains tested (OD_630_ range: 0.08–1.2). Most CF strains (80%) were moderate biofilm producers, whereas the majority of the NCF and NR strains (75%) were strong producers (Table 1). Strong biofilm producers exhibited increased swimming and twitching activity.

**FIGURE 1:**
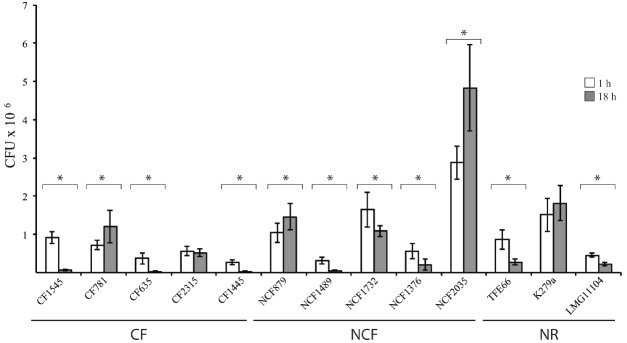
**Interactions of *S. maltophilia* strains with iDCs.** White and gray bar graphs indicate CFUs recovered by iDCs after 1 h and 18 h of infection, respectively. *p < 0.05. Each bar indicates the mean value ± standard deviation of at least three independent experiments in triplicate.

**FIGURE 2:**
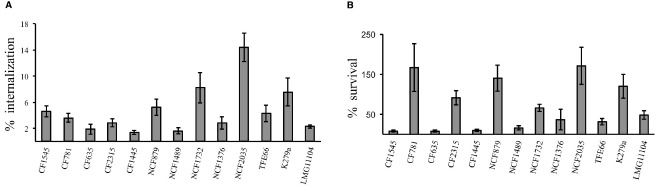
**DC infection assays. (A)** Entry Index (EI). Bar graphs shown the percentage internalization of *S. maltophilia* strains after 1 h of infection with DCs. **(B)** Intracellular Replication Index (RI). Bar graphs shown the percentage survival of *S. maltophilia* strains after 18 h of infection within DCs. Each bar indicates the mean value ± standard deviation of at least three independent experiments in triplicate.

The uptake and intracellular replication were analyzed by infecting immature monocytes-derived DCs (iDCs) with tested *S. maltophilia* strains at a MOI of 20. All strains were internalized and were able to survive within iDCs, but internalization and replication significantly varied among strains of the same group, as among strains of different groups (Figure 1). The difference between CFUs at 1 h and 18 h of infection resulted statistically significant (*p* < 0.05) for 11/13 strains (Figure 1). Values of Entry Index (EI) of CF strains ranged from 1.35 for (CF1445 strain) to 4.6 (CF1545 strain; Figure 2A). Only the CF781 strain was able to grow intracellularly (*p* < 0.05) with Intracellular Replication Index (RI) of 166.7 (Figure 2B). EI values of NCF strains ranged from 1,6 (NCF 1489 strain) to 14.38 (NCF2035 strain; Figure 2A). Two NCF isolates (NCF879 and NCF2035 strains) were able to multiply intracellularly (*p* < 0.05) with RI mean values of 140.38 and 170.83 respectively (Figure 2B). Among NR strains, only the K279a strain could survive into iDCs, but was unable to replicate, because no significant differences (*p* = 0,27) between CFUs at 1 h and 18 h were found (Figure 1). In spite of the limited number of clones analyzed, no significative difference could be observed among CF, NCF, and NR strains. This weakens the hypothesis made by several groups suggesting that unique properties may be associated to CF strains (see Waters et al., 2007).

### *S. maltophilia* Induces DCs Maturation

**FIGURE 3:**
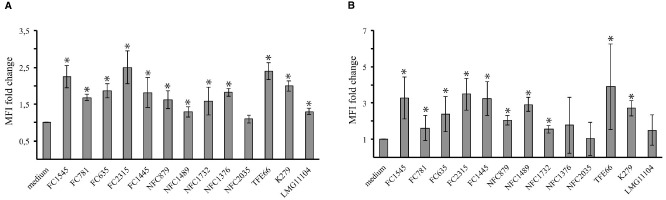
***Stenotrophomonas maltophilia* strains induce dendritic cells maturation.** Bar graphs shown the fold change in CD80 **(A)** and CD86 **(B)** expression after 18 h of incubation. CD86 and CD80 expression on DCs was measured by flow cytometry. The mean fluorescence intensity (MFI) reflects the upregulation of molecule on cell surface. Each bar indicates the mean ± standard deviation of three independent experiments. *p < 0.05. Medium: iDCs uninfected.

Changes induced in DCs by tested strains were assessed by measuring by flow cytometry the expression of CD86 and CD80, two costimulatory molecules upregulated during DC maturation. As revealed by the fold increase in mean fluorescence intensity (MFI) of CD80 and CD86 (Figure 3), most strains induced a consistent upregulation of both molecules. Only for the NCF2035 strain no statistically significative difference in the expression of both CD80 and CD86 in infected and uninfected cells could be observed.

**FIGURE 4:**
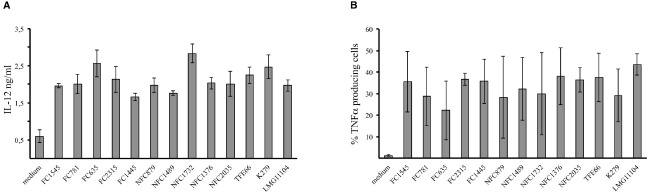
***Stenotrophomonas maltophilia* strains induce cytokines production by dendritic cells.** Bar graphs shown the production of IL-12 **(A)** and TNFα **(B)** by dendritic cells after incubation with the *S. maltophilia* strains. IL-12 and TNFα expression by DCs was measured by ELISA and flow cytometry, respectively. Each bar indicates mean ± standard deviation of at three independent experiments. All the bacterial strains induced the production of TNFα and IL-12 by DCs (*p* < 0.001). Medium: iDCs uninfected.

Along with changes in the expression of surface maturation markers, DC secrete several cytokines in response to intracellular infection. All strains induce a statistically significative (*p* < 0.001) increase of TNFα and IL-12 cytokines (Figure 4). Also in DCs maturation, no significant differences (*p* > 0.05) was observed by comparing CF isolates to either NCF or NR strains.

## Discussion

By sensing danger signals in peripheral tissues, iDCs activate both adaptive and innate immunity, leading to bacterial clearance. Several microbial pathogens are able to avoid or subvert DCs, thwarting DC differentiation, survival, maturation, antigen processing and presentation and the induction of T cell immunity (Rescigno, 2015). This is the first report of the interaction between human monocyte-derived DCs and *S. maltophilia*. All the analyzed strains were internalized by DCs (Figure 1), but the efficiency of internalization and intracellular survival differed highly (Figure 2). Six of thirteen strains exhibited low EI values, and were unable to persist inside host cells. Thus, these *S. maltophilia* isolates plausibly escape the effector cells of innate immunity by circumventing phagocytosis. Four strains showed high EI values, and two (NFC879 and NFC2035) also exhibited high RI values. NFC879- and NFC2035-like strains could safely be mobilized in the host evading antimicrobial therapy and immune response. The intracellular survival and replication could contribute to *S. maltophilia* virulence, and DCs may serve as dissemination vectors of bacteria outside of the infection site. Monitoring changes in gene expression occurred in bacteria recovered by DCs after 1 h and 18 h of infection could eventually help to clarify the intracellular survival strategy of *S. maltophilia* inside DCs.

Most of tested strains induced a significant increase in the expression of DC surface maturation markers (Figure 3). This suggests that DCs exposed to *S. maltophilia* can prime naïve T cells. Noteworthy, the NFC2035 strain, which exhibited the highest RI value, selectively failed to upregulate CD80 and CD86 costimulatory molecules. Pathogens as diverse as *Mycobacterium tuberculosis*, HIV-1, and *Leishmania* sp., downregulate the expression of costimulatory molecules in order to establish chronic infection. Impairment in the signaling events delivered by costimulatory molecules may be responsible for defective T-cell responses, enabling infective organisms to grow unhindered in the host cells (Khan et al., 2012).

All tested strains were able to induce the production of TNFα and IL-12 in DCs (Figure 4), and this is on line with data obtained by monitoring the response of macrophages to *S. maltophilia* (Waters et al., 2007). The production of both cytokines may play a crucial role in tissue inflammation, and contribute to a slow damage in CF lung. Clinical retrospective studies indeed showed deterioration of lung function after prolonged colonization with *S. maltophilia* in CF- as in hospital-acquired pneumonias (Karpati et al., 1994).

No significant difference was observed in the maturation of iDCs stimulated with *S. maltophilia* strains belonging to CF, NCF, and NR group. The data suggested that internalization, intracellular survival and immunostimulatory properties of *S. maltophilia* are strain-dependent. This is in accord with studies showing that different *S. maltophilia* strains replicate and persist in the murine lungs to significantly different degrees (Rouf et al., 2011). The observed heterogeneity is not surprising, in light of the high genetic diversity exhibited by *S. maltophilia* isolates (Roscetto et al., 2008; Kaiser et al., 2009; Rocco et al., 2009).

Further experiments will be needed to characterize the DC-driven T-cell responses, analyzing the ability of DCs infected with *S. maltophilia* to bias naive allogenic T-cell differentiation toward a TH_1_ or TH_2_ phenotype.

## Author Contributions

All authors read and approved this manuscript. ER and MC conceived and designed the experiments. ER, LV, RM, and AS performed the experiments. ER, LV, and EDG analyzed the data. VI and AV provided strains for analysis. The manuscript was prepared by ER, EDG, and MC.

### Conflict of Interest Statement

The authors declare that the research was conducted in the absence of any commercial or financial relationships that could be construed as a potential conflict of interest.
